# Awareness, treatment and control of cardiometabolic disorders in Chinese adults with diabetes: a national representative population study

**DOI:** 10.1186/s12933-015-0191-6

**Published:** 2015-02-26

**Authors:** Tiange Wang, Yu Xu, Min Xu, Weiqing Wang, Yufang Bi, Jieli Lu, Meng Dai, Di Zhang, Lin Ding, Baihui Xu, Jichao Sun, Wenhua Zhao, Yong Jiang, Limin Wang, Yichong Li, Mei Zhang, Shenghan Lai, Linhong Wang, Guang Ning

**Affiliations:** State Key Laboratory of Medical Genomics, Key Laboratory for Endocrine and Metabolic Diseases of Ministry of Health, National Clinical Research Center for Metabolic Diseases, and Shanghai Clinical Center for Endocrine and Metabolic Diseases, Department of Endocrine and Metabolic Diseases, Rui Jin Hospital, Shanghai Jiao Tong University School of Medicine, 197 Rui-Jin 2nd Road, Shanghai, 200025 China; National Center for Chronic and Noncommunicable Disease Control and Prevention, Chinese Center for Disease Control and Prevention, Beijing, China; Department of Pathology, Johns Hopkins University School of Medicine, Baltimore, MD USA

**Keywords:** Diabetes, Hypertension, Dyslipidemia, Control, China

## Abstract

**Background:**

The diagnosis of diabetes has important clinic implications for the prevention and management of cardiometabolic disorders. We aimed to investigate the awareness, treatment and control of hypertension and dyslipidemia in previously-diagnosed and newly-diagnosed diabetes in Chinese adult population.

**Methods:**

We conducted a cross-sectional survey in a nationally representative sample of 98658 Chinese adults aged 18 years or older in 2010, using a complex, multistage, probability sampling design. Glycemic status were defined according to the 2010 American Diabetes Association criteria. Hypertension was diagnosed by the Seventh Report of the Joint National Committee on Prevention, Detection, Evaluation, and Treatment of High Blood Pressure. Dyslipidemia was diagnosed by the 2004 National Cholesterol Education Program Adult Treatment Panel III.

**Results:**

The weighted prevalence of hypertension and dyslipidemia gradually increased in adults with normal glucose regulation, prediabetes, newly-diagnosed diabetes and previously-diagnosed diabetes. Compared to newly-diagnosed diabetes patients, previously-diagnosed diabetes patients were more likely to be aware of hypertension (weighted percentage [95% confidence interval]: 55.2% [52.9%-57.5%] vs 37.6% [35.9%-39.3%]) and dyslipidemia (33.9% [31.8%-36.1%] vs 12.8% [11.7%-13.9%]), to receive blood pressure-lowing (43.7% [41.5%-46.0%] vs 27.5% [26.0%-29.0%]) and lipid-lowering (18.9% [17.2%-20.7%] vs 5.4% [4.6%-6.2%]) therapies, and to have controlled blood pressure (4.7% [3.5%-6.2%] vs 3.5% [2.6%-4.8%]) and lipid (15.9% [12.3%-20.3%] vs 9.5% [6.4%-13.8%]) levels.

**Conclusions:**

Detection and control of hypertension and dyslipidemia is far from optimal in Chinese adults, especially in newly-diagnosed diabetes. Improved screening for diabetes is required to promote a better prevention, treatment and control of hypertension and dyslipidemia in China.

**Electronic supplementary material:**

The online version of this article (doi:10.1186/s12933-015-0191-6) contains supplementary material, which is available to authorized users.

## Background

Hypertension and dyslipidemia are established risk factors for cardiovascular disease morbidity and mortality [1]. Worldwide, 7.6 million premature deaths were attributed to high blood pressure in 2001 [2], and 4.4 million deaths each year were caused by raised cholesterols [3-6]. Most of the attributable burden caused by hypertension and dyslipidemia is borne by low-income and middle-income countries [2,7], where the coverage of screening and treatment of hypertension and dyslipidemia is low [6,8]. The awareness, treatment and control of hypertension and dyslipidemia have been discussed in several general populations [7,9,10]. Knowledge of the current magnitude of hypertension and dyslipidemia epidemic is key to ameliorating health care resource allocation, chronic disease management and education [11].

The China Noncommunicable Disease Surveillance 2010 is a nationally representative study [12], in which we have reported that 11.6% of Chinese adults aged 18 years or older had diabetes in 2010, while less than one-third (30.3%) of diabetes patients were aware of their condition [12]. Our data suggested that diabetes have reached an alert level in China, with the potential for a major epidemic of diabetes-related metabolic disorders, such as hypertension and dyslipidemia, which act as independent contributors to cardiovascular disease especially in patients with diabetes [13-15]. One previous study has indicated that newly-diagnosed diabetes tended to be associated with higher prevalence of uncontrolled hypertension and dyslipidemia [16]. Data from the United States National Health and Nutrition Examination Survey (NHANES) have revealed that newly-diagnosed diabetes was associated with a lack of awareness, treatment and control of high low-density lipoprotein cholesterol (LDL-C) [17]. Until recently, there are limited data concentrated on the awareness, treatment and control of hypertension and dyslipidemia in previously-diagnosed diabetes versus newly-diagnosed diabetes.

We sought to report a cross-sectional analysis of the influence of previously-diagnosed diabetes versus newly-diagnosed diabetes on the awareness, treatment and control of hypertension and dyslipidemia in a nationally representative sample from the China Noncommunicable Disease Surveillance 2010.

## Methods

### Study population

The China Noncommunicable Disease Surveillance 2010 included 162 study sites, based on the Chinese Center for Disease Control and Prevention’s National Disease Surveillance Point System, which guaranteed a nationally representative sample of the general population, covering major geographic areas of all 31 provinces, autonomous regions, and municipalities in mainland China [12,18]. The Surveillance Point System includes approximately 1% of the total Chinese population [18]. The first sampling level was stratified by 7 geographic regions including Northeast, North, East, South, Southeast, Northwest and Central areas, and 3 municipalities including Beijing, Tianjin, and Shanghai. The second level was stratified by urban and rural locations. The third level was stratified by 4 socioeconomic strata in rural areas and 3 population size strata in urban areas.

Only persons who had been living in their current residence for 6 months or longer were eligible to participate. At each site, a complex, multistage, probability sampling design was used to select participants who were representative of civilian, non-institutionalized Chinese adults. In stage one, 4 subdistricts in each urban area and 4 townships in each rural area were selected with probability proportional to population size. In stage two, 3 neighborhood communities or administrative villages were selected with probability proportional to population size. In stage three, households within each neighborhood community or administrative village were listed, and 50 households were randomly recruited. In stage four, 1 person aged 18 years or older was randomly selected from each household using a Kish selection table. When the selected individual was unable to participate, a similar household in the same or in a nearby neighborhood or village was randomly selected as a replacement. A total of 98658 adults (45143 men and 53515 women) participated in the survey. The overall response rate was 90.5%.

The study protocol was approved by the Ethical Review Committee of the Chinese Center for Disease Control and Prevention and other participating institutes. All study participants provided written informed consent.

### Data collection

Data were collected in examination centers at local health stations or community clinics in the participants’ residential area by investigators and staff, who were trained according to a standard protocol and were required to pass a performance test in order to be qualified for data collection. A questionnaire including demographic characteristics, medical history, and lifestyle factors was administered by trained interviewers with face-to-face interviews. Current smoking was defined as having smoked 100 cigarettes in one’s lifetime and currently smoking cigarettes. Current drinking was defined as consuming alcohol more than once per month during the past 12 months. Physical activity was assessed by the Global Physical Activity Questionnaire and was measured by the metabolic equivalent hours per week [19]. Body mass index (BMI) was calculated as body weight in kilograms divided by body height squared in meters (kg/m^2^). Waist circumference was measured on standing participants midway between the lower edge of the costal arch and the upper edge of the iliac crest. Overweight was defined as a BMI of 25.0-29.9 kg/m^2^, and obesity was defined as BMI ≥30.0 kg/m^2^. Central obesity was defined as waist circumference ≥90 cm in men and ≥80 cm in women. Participants were advised to avoid alcohol, coffee, tea, smoking and exercise at least 30 minutes before blood pressure measurement. Blood pressure was measured at the non-dominant arm 3 times consecutively with a 1-minute interval between the measurements of seated participants using a calibrated automatic electronic device (OMRON Model HEM-7071, Omron Co.) in a separate examination room after a 5-minute rest. The three readings were averaged for analysis.

Blood samples were collected in all participants after an overnight fast of 10 hours or more. Participants without a self-reported history of diabetes undertook a 75-g oral glucose tolerance test (OGTT), and plasma glucose was measured at 0 and 2 hours after glucose administration during the OGTT. Plasma glucose was measured locally using glucose oxidase or hexokinase methods within 24 hours. All study laboratories successfully completed a standardization and certification program. The Hemoglobin Capillary Collection System (Bio-Rad Laboratories) was used to collect capillary blood samples strictly according to the manufacturer’s instructions. The capillary blood specimens were shipped and stored at 2°C to 8°C until hemoglobin A1c (HbA1c) was measured within 4 weeks after collection by high-performance liquid chromatography using the VARIANT II Hemoglobin Testing System (Bio-Rad Laboratories) at the central laboratory in the Shanghai Institute of Endocrine and Metabolic Diseases, which was certificated by the National Glycohemoglobin Standardization Program. Capillary HbA1c was converted to venous values using a validated formula [12].

Serum samples were aliquoted and frozen at −80°C within 2 hours of collection and shipped by air in dry ice to the central laboratory, which was accredited by the College of American Pathologists. Serum total cholesterol, LDL-C, HDL-C, and triglycerides were measured using an autoanalyser (Abbott Laboratories).

### Definitions

Participants were categorized into one of the four mutually exclusive categories based on diabetes status: normal glucose regulation, prediabetes, newly-diagnosed diabetes, and previously-diagnosed diabetes, according to the American Diabetes Association 2010 criteria [20]. Diabetes was defined as a self-reported previous diagnosis by health care professionals, fasting plasma glucose level of 7.0 mmol/L (126 mg/dL) or higher, 2-hour plasma glucose level of 11.1 mmol/L (200 mg/dL) or higher, or HbA1c concentration of 6.5% or more. Among all diabetes patients, participants who reported a history of physician-diagnosed diabetes were considered to have previously-diagnosed diabetes, and those without a history of physician-diagnosed diabetes were considered to have newly-diagnosed diabetes. Prediabetes was defined as fasting plasma glucose levels between 5.6 mmol/L (100 mg/dL) and 6.9 mmol/L (125 mg/dL), 2-hour plasma glucose levels between 7.8 mmol/L (140 mg/dL) and 11.0 mmol/L (199 mg/dL), or HbA1c concentrations between 5.7% and 6.4% in participants without a prior diabetes diagnosis. Normal glucose regulation was defined as fasting plasma glucose levels less than 5.6 mmol/L (100 mg/dL), 2-hour plasma glucose levels less than 7.8 mmol/L (140 mg/dL), and HbA1c concentrations less than 5.7% in participants without a prior diabetes diagnosis.

The diagnosis and categorization of hypertension were based on the Seventh Report of the Joint National Committee on Prevention, Detection, Evaluation, and Treatment of High Blood Pressure [21]. Hypertension was defined as a systolic blood pressure of 140 mm Hg or higher, a diastolic blood pressure of 90 mmHg or higher or taking blood pressure-lowering medications within the previous 2 weeks. Awareness was defined as the proportion of participants who reported a history of physician-diagnosed hypertension among all patients with hypertension. Treatment was defined as the proportion of participants taking blood pressure-lowering medications among all patients with hypertension. Control was defined as the proportion of participants who had systolic blood pressure and diastolic blood pressure of less than 140/90 mmHg for non-diabetes participants, or of less than 130/80 mmHg for diabetes patients among all hypertension patients with blood pressure-lowering medications.

According to the 2004 National Cholesterol Education Program Adult Treatment Panel III [22], dyslipidemia was defined as taking lipid-lowering medications or increased lipid levels as total cholesterol ≥240 mg/dL, triglycerides ≥200 mg/dL, HDL-C <40 mg/dL, or high LDL-C. Framingham risk score was calculated to estimate the 10-year risk of coronary heart disease [23]. Coronary heart disease risk factors included older age (55 years or older for women and 45 years or older for men), current smoking, hypertension, and low HDL-C (less than 40 mg/dL). Participants were assigned to one of the four risk categories: 1) low risk, having 0 to 1 risk factor, 2) intermediately low risk, having 2 or more risk factors and a 10-year risk of coronary heart disease less than 10%, 3) intermediately high risk, having 2 or more risk factors and a 10-year risk of coronary heart disease between 10% and 20%, and 4) with diabetes or a 10-year risk of coronary heart disease greater than 20% [24]. High LDL-C was defined according to the group-specific threshold recommended in National Cholesterol Education Program Adult Treatment Panel III as ≥190, 160, 130 and 130 mg/dL for risk categories 1 through 4, respectively. Awareness was defined as the proportion of participants who reported a history of physician-diagnosed dyslipidemia among all patients with dyslipidemia. Treatment was defined as the proportion of participants taking lipid-lowering medications among all patients with dyslipidemia. Control was defined as the proportion of participants who had total cholesterol <240 mg/dL, triglycerides <150 mg/dL, HDL-C ≥40 mg/dL in men and ≥50 mg/dL in women, and LDL-C <160, 130, 100, and 100 mg/dL for risk categories 1 through 4, respectively, among all patients with lipid-lowering medications.

### Statistical analysis

All calculations were weighted to represent the overall Chinese adult population aged 18 years or older. Weight coefficients were derived from China population census data in 2010 and the sampling scheme of the present survey to obtain national estimates. Each one of the 162 study sites was categorized into underdeveloped, intermediately developed or developed region according to their gross domestic product per capita in 2009.

Demographic and metabolic features were described in overall population and in diabetes categories, using percentages (95% confidence intervals [CIs]) for categorical variables and means (95% CIs) for continuous variables. Weighted percentages (95% CIs) for prevalence, awareness, treatment and control of hypertension and dyslipidemia were estimated in overall population and in different diabetes categories.

Data were analyzed using the SAS system, version 9.3 (SAS Institute Inc, Cary, NC) and SUDAAN software, version 10.0 (Research Triangle Institute, Research Triangle Park, NC). All statistical analyses were 2-sided, and a *P*-value less than 0.05 was considered statistically significant.

## Results

Among Chinese adults aged 18 years or older, the prevalence of diabetes was estimated to be 11.6% (95% CI: 11.3%, 11.8%), among which 3.5% (95% CI: 3.4%, 3.6%) were previously-diagnosed diabetes and 8.1% (95% CI: 7.9%, 8.3%) were newly-diagnosed diabetes. Characteristics of study population by glycemic status are depicted in Table 1. Compared with newly-diagnosed diabetes patients, previously-diagnosed diabetes patients were older, more likely to be women, or urban residents, live in economically developed areas, have parental diabetes history and higher educational achievement, have lower proportions of current smokers or drinkers, and have less physical activity. Additionally, previously-diagnosed diabetes patients had higher levels of BMI, waist circumference, fasting plasma glucose, HbA1c, and systolic blood pressure, and lower levels of OGTT-2 h plasma glucose, total cholesterol, triglycerides, LDL-C, and HDL-C than newly-diagnosed diabetes patients.

**Table 1:** **Characteristics of Chinese adults by glycemic status**

	**Glycemic status**			
	**Normal glucose regulation**	**Prediabetes**	**Newly-diagnosed diabetes**	**Previously-diagnosed diabetes**
Age, year	37.3 (37.1, 37.5)	44.9 (44.7, 45.2)	50.6 (50.1, 51.1)	54.9 (54.3, 55.5)
Sex				
Men	47.4 (46.7, 48.2)	52.7 (52.1, 53.3)	53.3 (51.9, 54.7)	52.5 (50.6, 54.4)
Women	52.6 (51.9, 53.3)	47.3 (46.7, 47.9)	46.7 (45.3, 48.1)	47.5 (45.6, 49.4)
Location				
Urban	30.1 (29.7, 30.6)	30.2 (29.8, 30.5)	33.8 (32.7, 35.0)	49.9 (48.0, 51.8)
Rural	69.9 (69.4, 70.4)	69.8 (69.5, 70.2)	66.2 (65.0, 67.4)	50.1 (48.2, 52.0)
Economic development				
Underdeveloped	32.7 (32.1, 33.3)	35.8 (35.4, 36.2)	32.9 (31.5, 34.2)	19.9 (18.4, 21.6)
Intermediately developed	36.3 (35.7, 36.8)	31.7 (31.3, 32.1)	30.2 (29.0, 31.5)	30.9 (29.2, 32.6)
Developed	31.0 (30.5, 31.5)	32.5 (32.1, 32.9)	36.9 (35.7, 38.2)	49.2 (47.3, 51.1)
Parental diabetes	4.7 (4.4, 5.0)	5.1 (4.8, 5.3)	6.8 (6.2, 7.6)	21.4 (19.8, 23.1)
Junior high education or more	69.2 (68.6, 69.8)	58.3 (57.7, 58.8)	54.1 (52.7, 55.5)	58.1 (56.3, 60.0)
Current smoking	26.9 (26.3, 27.5)	29.7 (29.2, 30.3)	28.6 (27.4, 29.9)	24.0 (22.3, 25.7)
Current drinking	28.2 (27.6, 28.8)	31.0 (30.4, 31.5)	30.1 (28.9, 31.5)	24.2 (22.6, 26.0)
Physical activity, MET-h/wk	85.2 (83.7, 86.7)	92.3 (91.0, 93.6)	81.1 (78.2, 84.0)	61.0 (58.2, 63.8)
BMI, kg/m^2^	22.9 (22.9, 23.0)	24.0 (23.9, 24.0)	25.3 (25.2, 25.5)	25.5 (25.4, 25.7)
Waist circumference, cm	77.6 (77.5, 77.8)	81.0 (80.8, 81.1)	85.7 (85.3, 86.0)	86.5 (86.1, 87.0)
Fasting plasma glucose, mmol/L	4.9 (4.9, 4.9)	5.6 (5.6, 5.6)	7.6 (7.5, 7.6)	8.7 (8.6, 8.9)
OGTT-2h plasma glucose, mmol/L	5.3 (5.2, 5.3)	6.2 (6.2, 6.3)	11.0 (10.9, 11.2)	9.7 (9.0, 10.4)
HbA1c, %	5.4 (5.4, 5.4)	5.8 (5.8, 5.8)	6.9 (6.8, 6.9)	7.8 (7.7, 7.8)
Systolic blood pressure, mmHg	125.6 (125.4, 125.9)	133.7 (133.4, 133.9)	143.4 (142.7, 144.1)	146.0 (145.1, 146.9)
Diastolic blood pressure, mmHg	78.2 (78.0, 78.3)	81.7 (81.5, 81.8)	86.0 (85.6, 86.3)	86.4 (86.0, 86.9)
Total cholesterol, mg/dL	146.8 (146.3, 147.3)	161.8 (161.3, 162.3)	176.7 (175.3, 178.1)	174.3 (172.5, 176.2)
Triglycerides, mg/dL	102.5 (101.2, 103.7)	124.4 (123.0, 125.8)	181.0 (176.0, 187.7)	179.9 (172.6, 187.1)
LDL-C, mg/dL	80.8 (80.5, 81.2)	91.9 (91.5, 92.2)	100.4 (99.5, 101.4)	99.8 (98.5, 101.0)
HDL-C, mg/dL	43.0 (42.8, 43.1)	42.9 (42.8, 43.1)	41.5 (41.2, 41.9)	40.0 (39.6, 40.5)

Data are weighted means (95% CIs) for continuous variables, and weighted percentages (95% CIs) for categorical variables.

*Abbreviations*: CI, confidence interval; MET-h/wk, metabolic equivalent hours per week; BMI, body mass index; OGTT, oral glucose tolerance test; HbA1c, hemoglobin A1c; LDL-C, low-density lipoprotein cholesterol; HDL, high-density lipoprotein cholesterol.

The weighted prevalence of hypertension and dyslipidemia gradually increased in adults with normal glucose regulation, prediabetes, newly-diagnosed diabetes and previously-diagnosed diabetes. Compared to those with newly-diagnosed diabetes, previously-diagnosed diabetes patients were more likely to be aware of hypertension (55.2% [95% CI: 52.9%, 57.5%] in previously-diagnosed diabetes and 37.6% [95% CI: 35.9%, 39.3%] in newly-diagnosed diabetes) and dyslipidemia (33.9% [95% CI: 31.8%, 36.1%] in previously-diagnosed diabetes and 12.8% [95% CI: 11.7%, 13.9%] in newly-diagnosed diabetes). Among participants who were aware of their conditions, those with previously-diagnosed diabetes were more inclined to receive treatment than those with newly-diagnosed diabetes (anti-hypertensive treatment, 43.7% [95% CI: 41.5%, 46.0%] in previously-diagnosed diabetes and 27.5% [95% CI: 26.0%, 29.0%] in newly-diagnosed diabetes; lipid-lowering treatment, 18.9% [95% CI: 17.2%, 20.7%] in previously-diagnosed diabetes and 5.4% [95% CI: 4.6%, 6.2%] in newly-diagnosed diabetes). The proportions of participants having controlled blood pressure and lipid levels were higher in those with previously-diagnosed diabetes (blood pressure control, 4.7% [95% CI: 3.5%, 6.2%]; lipid control, 15.9% [95% CI: 12.3%, 20.3%]) than in those with newly-diagnosed diabetes (blood pressure control, 3.5% [95% CI: 2.6%, 4.8%]; lipid control, 9.5% [95% CI: 6.4%, 13.8%]) (Table 2).

**Table 2:** **Awareness, treatment and control of hypertension and dyslipidemia in Chinese adults by glycemic status**

	**Glycemic status**			
	**Normal glucose regulation**	**Prediabetes**	**Newly-diagnosed diabetes**	**Previously-diagnosed diabetes**
Hypertension				
Prevalence	21.9 (21.3, 22.4)	37.1 (36.6, 37.7)	56.8 (55.4, 58.2)	66.3 (64.5, 68.1)^a^
Awareness	28.4 (27.3, 29.6)	32.0 (31.2, 32.8)	37.6 (35.9, 39.3)	55.2 (52.9, 57.5)^a^
Treatment	19.2(18.2, 20.2)	22.8 (22.1, 23.5)	27.5 (26.0, 29.0)	43.7 (41.5, 46.0)^a^
Control	19.6 (17.5, 21.9)	15.7 (14.6, 17.0)	3.5 (2.6, 4.8)	4.7 (3.5, 6.2)
Dyslipidemia				
Prevalence	47.2 (46.5, 47.9)	51.5 (50.9, 52.1)	63.2 (61.8, 64.5)	70.0 (68.2, 71.7)^a^
Awareness	5.4 (5.0, 5.8)	8.3 (7.9, 8.7)	12.8 (11.7, 13.9)	33.9 (31.8, 36.1)^a^
Treatment	2.3 (2.0, 2.5)	3.7 (3.5, 4.0)	5.4 (4.6, 6.2)	18.9 (17.2, 20.7)^a^
Control	20.1 (15.8, 25.2)	15.6 (13.1, 18.5)	9.5 (6.4, 13.8)	15.9 (12.3, 20.3)
Total cholesterol control	94.3 (90.8, 96.5)	91.3 (89.1, 93.1)	89.3 (84.4, 92.8)	94.1 (91.5, 96.0)
LDL-C control	94.7 (91.6, 96.7)	88.2 (85.9, 90.2)	77.9 (71.1, 83.5)	87.4 (83.7, 90.3)^b^
HDL-C control	28.2 (23.3, 33.6)	29.3 (26.0, 32.9)	27.2 (21.4, 34.0)	26.8 (22.4, 31.8)
Triglycerides control	61.3 (55.6, 66.7)	53.9 (50.1, 57.6)	44.2 (37.1, 51.5)	50.2 (45.0, 55.4)

Data are weighted percentages (95% CIs).

^a^P < 0.0001, ^b^P < 0.05, compared with newly-diagnosed diabetes.

As shown in Table 3, the proportions of awareness and treatment of hypertension were higher in individuals who were older, women, urban residents, living in economically developed areas, and with higher BMI and abdominal obesity in both newly-diagnosed and previously-diagnosed diabetes. Compared with newly-diagnosed diabetes, previously-diagnosed diabetes had higher proportions of awareness and treatment of hypertension and dyslipidemia in each stratum, had higher proportions of controlled blood pressure in 45–64 years old individuals, and had higher proportions of controlled lipids in 45–64 years old individuals, men, urban residents, those with BMI less than 25 kg/m^2^, and those with waist circumference <90 cm in men and <80 cm in women (Table 4).

**Table 3:** **Awareness, treatment and control of hypertension in Chinese adults with previously-diagnosed diabetes and newly-diagnosed diabetes**

	**Newly-diagnosed diabetes**			**Previously-diagnosed diabetes**		
	**Awareness**	**Treatment**	**Control**	**Awareness**	**Treatment**	**Control**
Age groups, year						
18-44	21.8 (18.6, 25.4)	11.5 (9.2, 14.3)	2.1 (0.7, 5.9)	38.6 (32.4, 45.1)^a^	25.8 (20.7, 31.8)^a^	5.4 (2.3, 12.5)
45-64	40.9 (38.9, 43.2)	29.9 (27.9, 32.0)	3.8 (2.5, 5.8)	55.0 (52.0, 58.0)^a^	43.6 (40.7, 46.5)^a^	4.2 (2.7, 6.3)^b^
≥65	45.2 (41.9, 48.6)	36.9 (33.8, 40.2)	3.5 (2.1, 5.8)	64.6 (60.6, 68.4)^a^	53.8 (49.7, 57.8)^a^	5.2 (3.3, 8.0)
Sex						
Men	33.9 (31.5, 36.3)	22.4 (20.4, 24.5)	3.5 (2.0, 6.0)	52.4 (48.9, 55.9)^a^	39.2 (36.0, 42.6)^a^	5.1 (3.4, 7.6)
Women	41.6 (39.2, 44.1)	33.0 (30.8, 35.3)	3.6 (2.5, 5.1)	58.1 (55.0, 61.1)^a^	48.4 (45.3, 51.4)^a^	4.4 (2.9, 6.6)
Location						
Urban	41.8 (39.3, 44.3)	33.2 (30.9, 35.6)	5.7 (3.9, 8.3)	59.2 (56.2, 62.1)^a^	48.6 (45.7, 51.6)^a^	6.5 (4.8, 8.8)
Rural	35.2 (33.0, 37.5)	24.3 (22.4, 26.3)	1.9 (1.1, 3.2)	51.4 (47.9, 54.9)^a^	39.1 (35.8, 42.4)^a^	2.5 (1.3, 4.9)
Economic development						
Underdeveloped	33.3 (30.1, 36.7)	23.6 (20.8, 26.6)	1.8 (0.7, 4.3)	48.4 (42.7, 54.1)^a^	37.3 (32.0, 42.9)^a^	4.9 (2.2, 10.9)
Intermediately developed	36.7 (33.9, 39.7)	25.3 (22.8, 28.0)	2.7 (1.5, 4.8)	52.1 (47.9, 56.3)^a^	39.9 (36.0, 43.9)^a^	1.9 (1.0, 3.5)
Developed	41.5 (38.9, 44.2)	32.2 (29.8, 34.7)	5.0 (3.4, 7.4)	60.0 (56.6, 62.7)^a^	48.5 (45.5, 51.6)^a^	6.0 (4.3, 8.4)
Body mass index, kg/m^2^						
<25.0	32.8 (30.2, 35.6)	23.4 (21.1, 25.9)	4.1 (2.6, 6.4)	52.2 (48.4, 55.9)^a^	41.3 (37.7, 44.9)^a^	7.5 (5.0, 11.2)
25.0-29.9	40.7 (38.2, 43.3)	29.3 (27.1, 31.6)	3.7 (2.3, 5.8)	56.4 (53.1, 59.6)^a^	45.0 (41.8, 48.3)^a^	3.3 (2.2, 5.0)
≥30.0	41.1 (36.9, 45.5)	33.0 (29.1, 37.2)	2.2 (0.8, 5.7)	60.6 (53.8, 67.0)^a^	46.7 (40.3, 53.2)^a^	1.8 (0.6, 5.1)
Waist circumference, cm						
<90 in men, < 80 in women	32.1 (29.5, 34.9)	21.0 (18.9, 23.4)	4.8 (2.8, 8.0)	50.7 (46.7, 54.7)^a^	37.9 (34.2, 41.8)^a^	8.0 (5.2, 12.0)
≥90 in men, ≥ 80 in women	40.9 (38.8, 43.1)	31.5 (29.5, 33.5)	3.0 (2.0, 4.5)	57.8 (55.0, 60.6)^a^	47.1 (44.4, 49.9)^a^	3.2 (2.1, 4.6)

Data are weighted percentages (95% CIs). ^a^P < 0.0001, ^b^P < 0.05, compared with newly-diagnosed diabetes.

**Table 4:** **Awareness, treatment and control of dyslipidemia in Chinese adults with previously-diagnosed diabetes and newly-diagnosed diabetes**

	**Newly-diagnosed diabetes**			**Previously-diagnosed diabetes**		
	**Awareness**	**Treatment**	**Control**	**Awareness**	**Treatment**	**Control**
Age groups, year						
18-44	8.2 (6.5, 10.3)	3.1 (2.0, 4.7)	-	26.1 (21.6, 31.0)^a^	10.6 (7.7, 14.5)^a^	12.3 (4.3, 30.9)
45- 64	15.3 (13.8, 17.0)	6.1 (5.1, 7.2)	12.1 (7.4, 19.1)	38.4 (35.5, 41.3)^a^	22.3 (19.9, 24.9)^a^	17.6 (13.0, 23.5)^b^
≥65	15.7 (13.3, 18.4)	8.1 (6.3, 10.4)	12.1 (6.5, 21.3)	32.7 (28.8, 36.7)^a^	20.2 (17.0, 23.8)^a^	14.0 (8.5, 22.1)
Sex						
Men	12.1 (10.6, 13.7)	4.2 (3.3, 5.3)	10.5 (6.1, 17.5)	32.4 (29.4, 35.4)^a^	16.6 (14.3, 19.1)^a^	19.6 (13.8, 27.0)^b^
Women	13.7 (12.2, 15.3)	6.8 (5.7, 8.1)	8.7 (4.9, 14.9)	35.7 (32.8, 38.7)^a^	21.6 (19.2, 24.2)^a^	12.5 (8.6, 17.8)
Location						
Urban	18.0 (16.2, 19.9)	6.1 (5.0, 7.3)	6.8 (3.6, 12.5)	36.3 (33.5, 39.2)^a^	17.7 (15.6, 20.0)^a^	17.1 (12.0, 23.7)^b^
Rural	9.8 (8.6, 11.3)	5.0 (4.0, 6.1)	11.3 (6.9, 18.0)	31.4 (28.4, 34.6)^a^	20.0 (17.5, 22.8)^a^	14.8 (10.2, 21.0)
Economic development						
Underdeveloped	6.6 (5.2, 8.3)	3.3 (2.3, 4.8)	10.2 (3.6, 25.7)	23.8 (19.7, 28.5)^a^	13.7 (10.7, 17.3)^a^	25.9 (15.3, 40.5)
Intermediately developed	12.7 (10.7, 14.9)	6.9 (5.3, 8.8)	10.3 (5.7, 18.0)	36.6 (32.8, 40.6)^a^	23.1 (19.8, 26.7)^a^	12.7 (8.0, 19.6)
Developed	18.0 (16.1, 20.0)	5.8 (4.8, 7.1)	8.3 (4.6, 14.5)	36.3 (33.3, 39.3)^a^	18.4 (16.1, 20.9)^a^	15.3 (10.4, 21.9)
Body mass index, kg/m^2^						
<25.0	7.7 (6.3, 9.4)	3.8 (2.7, 5.2)	10.5 (5.2, 20.0)	26.6 (23.6, 29.8)^a^	15.9 (13.5, 18.7)^a^	22.0 (15.5, 30.3)^b^
25.0-29.9	15.7 (14.0, 17.5)	6.3 (5.2, 7.5)	11.2 (6.8, 17.9)	37.2 (34.1, 40.4)^a^	20.6 (18.1, 23.4)^a^	13.3 (8.7, 19.7)
≥30.0	17.8 (14.8, 21.3)	6.8 (5.1, 9.2)	2.5 (0.6, 10.1)	46.2 (40.0, 52.9)^a^	21.9 (17.2, 27.5)^a^	10.4 (4.6, 21.5)
Waist circumference, cm						
<90 in men, < 80 in women	8.3 (6.8, 10.0)	3.5 (2.4, 4.9)	12.2 (6.2, 22.7)	26.8 (23.5, 30.2)^a^	14.6 (12.1, 17.4)^a^	25.8 (18.2, 35.2)^b^
≥90 in men, ≥ 80 in women	15.7 (14.3, 17.3)	6.6 (5.7, 7.7)	8.6 (5.2, 13.7)	38.5 (35.8, 41.2)^a^	21.6 (19.4, 24.0)^a^	11.6 (8.0, 16.7)

Data are weighted percentages (95% CIs). ^a^P < 0.0001, ^b^P < 0.05, compared with newly-diagnosed diabetes.

**Figure 1:**
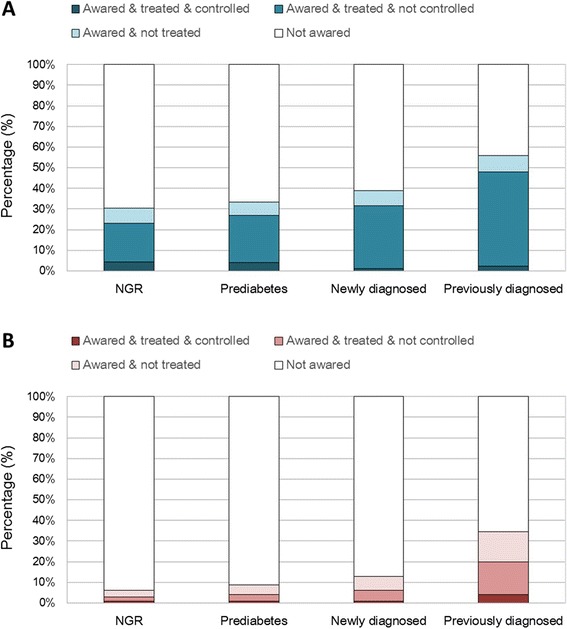
**Awareness, treatment and control of hypertension (A) and dyslipidemia (B) by glycemic status.**

Among all participants, the proportion of participants who were aware of hypertension, in the meanwhile received anti-hypertensive medications and had their blood pressure controlled was 2.1% (95% CI: 1.5%, 2.7%) in previously-diagnosed diabetes patients vs newly-diagnosed diabetes (1.0% [95% CI: 0.7%, 1.3%]). Treatment and control of dyslipidemia were even worse than that of hypertension. The proportion of controlled lipids and treated dyslipidemia was higher in previously-diagnosed diabetes (3.0%, 95% CI: 2.3%, 3.9%) than newly-diagnosed diabetes (0.5%, 95% CI: 0.3%, 0.8%), as well as the proportion of treated but uncontrolled patients (Figure 1).

## Discussion

In this nationally representative survey of Chinese adults aged 18 years or older, we found that previously-diagnosed diabetes was associated with a higher likelihood of awareness, treatment and control of hypertension and dyslipidemia compared to newly-diagnosed diabetes. Especially, a significant proportion of newly-diagnosed diabetes patients remained unaware of hypertension and dyslipidemia, and only a minority were treated and had their conditions effectively controlled.

The present study was conducted in a large nationally representative sample of the general population in China. In addition, the diagnosis of diabetes used all 3 glycemic indexes including fasting plasma glucose, 2-hour plasma glucose, and HbA1c concentrations, which provide a comprehensive estimation of diabetes status. Moreover, a strict quality assurance and quality control program was implemented at every phase of the study to ensure data validity and reliability. Several study limitations should be mentioned. First, type 1 and type 2 diabetes were not distinguished, although type 2 diabetes is the predominant diabetes in adults. Second, correlations may not infer causal relationships because of the cross-sectional nature and potential reverse causation bias. Also, the cross-sectional nature of the study prevents from seeking more deeply the reasons why subjects with previously diagnosed type 2 diabetes showed greater level of awareness of concomitant risk factors such as hypertension and dyslipidemia, compared to newly diagnosed subjects. In addition, due to a lack of family history of coronary heart disease (history of myocardial infarction or angina before age 50 years among first-degree relatives), we cannot precisely define the coronary heart disease risk categories.

Our data elaborated that not only in overall Chinese population, but also in traditional risk factors’ strata, previously-diagnosed diabetes patients were more likely to be aware of hypertension and dyslipidemia and to receive therapy. This result can be partially explained by the fact that compared to newly-diagnosed diabetic patients, previously-diagnosed patients are more frequently to have medical examinations by physicians. As a result of the diagnosis of diabetes, some hypertension and dyslipidemia could be prompted to have treated and get controlled. Thus, screening for diabetes would make for early diagnosis and prevention of hypertension and dyslipidemia, which would benefit the prevention and control of atherosclerotic cardiovascular diseases in general population [25-27].

The diagnosis of diabetes has important clinic implications for the prevention and management of cardiometabolic complications. The Ludwigshafen Risk and Cardiovascular Health study found that half of the patients scheduled for coronary angiography had newly-diagnosed diabetes, suggesting that for high-risk patients who had to schedule for coronary angiography, screening for diabetes should be performed routinely to initiate timely preventive efforts [28]. Besides, newly-diagnosed diabetes was common among high-risk non-ST-segment elevation acute coronary syndrome patients and associated with greater 30-day death or myocardial infarction [29], and was also associated with a higher death rate after coronary artery bypass grafting [30].

Hypertension and hyperlipidemia increase the risk of long-term cardiovascular disease in type 2 diabetes [31,32], which are disproportionally more harmful in low- and middle-income countries than in high-income countries [2,7]. In our study, urban location, developed economy, high education, and parental diabetes were associated with higher rate of hypertension control in both previously-diagnosed and newly-diagnosed diabetes. Urban location, high education, and parental diabetes were associated with higher proportion of controlled blood lipids in previously-diagnosed diabetes, but not in newly-diagnosed diabetes. Even in previously-diagnosed diabetes patients who were aware of their conditions and received treatments, the control rates for hypertension and dyslipidemia were still low. Several risk factors, including old age, women, rural location, under-developed economy, without parental history of diabetes, low education, less physical activity, high glucose or HbA1c associated with the worse control of hypertension in previously-diagnosed diabetes patients. These risk factors also impact the control of dyslipidemia, except that the developed economy was associated with lower control of dyslipidemia, and suggests that preventive interventions for dyslipidemia should be used at all levels of economic development (Additional file 1: Table S1 and S2). Interestingly, in each glycemic status, compared to non-obese individuals, general or abdominal obese individuals had higher prevalence of hypertension and dyslipidemia, and were more likely to be aware and treated, whereas were less likely to be adequately controlled (Additional file 1: Figure S1). The increased prevalence of obesity may have not only affected the metabolic characteristics of the population, but also impeded the pursuit of diseases control [33,34]. Thus, controlling obesity is important for an efficient and comprehensive management of multiple risk factors to prevent atherosclerotic cardiovascular diseases [35].

## Conclusions

The China Noncommunicable Disease Surveillance 2010 indicated that the detection and control of hypertension and dyslipidemia is far from optimal in Chinese adults, especially in newly-diagnosed diabetes. Our data suggest that improved screening for diabetes is required to be an important component of a national strategy to promote better prevention, treatment and control of hypertension and hyperlipidemia, and to ease the large and increasing societal burden of cardiovascular disease in China.

## Additional file

Additional file 1: Table S1. Characteristics of Chinese adults with controlled vs. uncontrolled hypertension in newly-diagnosed and previously-diagnosed diabetes. **Table S2.** Characteristics of Chinese adults with controlled vs. uncontrolled dyslipidemia in newly-diagnosed and previously-diagnosed diabetes. **Figure S1.** Prevalence, awareness, treatment and control of hypertension and dyslipidemia by obesity status in each glycemic group.

## Abbreviations

BMI: Body mass index
CI: Confidence interval
HbA1c: Hemoglobin A1c
HDL: High-density lipoprotein cholesterol
LDL-C: Low-density lipoprotein cholesterol
MET-h/wk: Metabolic equivalent hours per week
NHANES: National Health and Nutrition Examination Survey
OGTT: Oral glucose tolerance test
