# Supplementary material for: Awareness, treatment and control of cardiometabolic disorders in Chinese adults with diabetes: a national representative population study
Source: Cardiovasc Diabetol. 2015 Feb 26;14:28. doi: 10.1186/s12933-015-0191-6 (PMC4356058; doi:10.1186/s12933-015-0191-6)

**Additional file 1**

**Table S1. Characteristics of Chinese adults with controlled vs. uncontrolled hypertension in newly-diagnosed and previously-**diagnosed diabetes

|  | Newly-diagnosed diabetes | |  | Previously-diagnosed diabetes | |
| --- | --- | --- | --- | --- | --- |
|  | Aware and treated and controlled | Aware and treated but not controlled |  | Aware and treated and  controlled | Aware and treated but not controlled |
| Age, year | 60.6 (57.3, 63.8) | 60.0 (59.1, 60.9) |  | 59.3 (55.0, 63.7) | 60.4 (59.5, 61.3) |
| Men | 40.8 (27.1, 56.1) | 43.3 (40.3, 46.4) |  | 47.0 (34.0, 60.4) | 44.9 (41.7, 48.2) |
| Urban location | 55.8 (40.9, 69.7) | 39.0 (36.5, 41.7) |  | 69.1 (54.7, 80.6) | 52.1 (49.0, 55.1) |
| Economically developed | 53.9 (39.2, 67.9) | 44.1 (41.4, 46.9) |  | 64.3 (50.7, 76.0) | 54.8 (51.8, 57.9) |
| Parental diabetes | 12.0 (4.7, 27.3) | 7.0 (5.7, 8.6) |  | 26.4 (16.5, 39.6) | 19.6 (17.1, 22.4) |
| Junior high education or more | 54.9 (40.3, 68.7) | 43.2 (40.2, 46.2) |  | 64.1 (50.6, 75.7) | 51.2 (48.0, 54.4) |
| Current smoking | 32.2 (19.2, 48.6) | 22.4 (19.9, 25.1) |  | 20.7 (12.2, 32.8) | 18.8 (16.3, 21.6) |
| Current drinking | 18.2 (10.0, 30.9) | 23.7 (21.2, 26.5) |  | 25.2 (14.8, 39.3) | 19.5 (17.0, 22.3) |
| Physical activity, MET, h/wk | 44.0 (32.7, 55.3) | 65.8 (60.6, 71.0) |  | 63.8 (39.0, 88.5) | 54.6 (50.1, 59.1) |
| BMI, kg/m^2^ | 25.9 (24.7, 27.1) | 26.8 (26.5, 27.1) |  | 24.6 (23.6, 25.5) | 26.5 (26.2, 26.7) |
| Waist circumference, cm | 86.6 (83.4, 89.8) | 89.7 (89.0, 90.4) |  | 83.3 (80.3, 86.3) | 89.1 (88.5, 89.7) |
| Fasting plasma glucose, mmol/L | 7.1 (6.4, 7.7) | 7.4 (7.3, 7.5) |  | 7.9 (7.1, 8.8) | 8.3 (8.1, 8.5) |
| OGTT-2h plasma glucose, mmol/L | 10.1 (8.6, 11.6) | 11.9 (11.6, 12.2) |  | 6.5 (4.8, 8.2) | 9.6 (8.4, 10.9) |
| Hemoglobin A1c, % | 6.6 (6.3, 6.8) | 6.9 (6.8, 7.0) |  | 7.2 (6.8, 7.7) | 7.5 (7.4, 7.7) |
| Systolic blood pressure, mmHg | 123.2 (121.6, 124.8) | 164.3 (162.9, 165.8) |  | 120.5 (118.5, 122.6) | 160.6 (159.2, 161.9) |
| Diastolic blood pressure, mmHg | 72.7 (70.8, 74.5) | 94.1 (93.3, 95.0) |  | 74.3 (73.2, 75.4) | 91.6 (90.8, 92.4) |
| Total cholesterol, mg/dL | 165.5 (154.6, 176.3) | 183.2 (180.0, 186.4) |  | 172.4 (159.9, 184.9) | 178.2 (175.2, 181.1) |
| Triglycerides, mg/dL | 115.2 (96.0, 134.5) | 185.8 (175.0, 196.6) |  | 137.8 (103.4, 172.3) | 184.8 (172.2, 197.4) |
| LDL-C, mg/dL | 97.7 (90.0, 105.4) | 106.1 (103.8, 108.4) |  | 97.8 (89.1, 106.4) | 102.4 (100.2, 104.6) |
| HDL-C, mg/dL | 42.2 (38.4, 46.1) | 40.9 (40.1, 41.6) |  | 43.9 (41.2, 46.6) | 39.8 (39.1, 40.5) |

Data are weighted means (95% CIs) for continuous variables, and weighted percentages (95% CIs) for categorical variables.

Abbreviations: MET, metabolic equivalent; BMI, body mass index; OGTT, oral glucose tolerance test; LDL, C, low, density lipoprotein cholesterol; HDL, C, high, density lipoprotein cholesterol.

**Table S2. Characteristics of Chinese adults with controlled vs. uncontrolled dyslipidemia in newly-diagnosed and previously-diagnosed diabetes**

|  | Newly-diagnosed diabetes | |  | Previously-diagnosed diabetes | |
| --- | --- | --- | --- | --- | --- |
|  | Aware and treated and controlled | Aware and treated but not controlled |  | Aware and treated and controlled | Aware and treated but not controlled |
| Age, year | 61.0 (58.3, 63.6) | 54.5 (51.5, 57.5) |  | 56.3 (52.9, 59.6) | 57.4 (56.0, 58.8) |
| Men | 25.2 (11.4, 47.1) | 44.6 (36.8, 52.5) |  | 52.7 (37.4, 67.4) | 47.2 (41.8, 52.8) |
| Urban location | 17.2 (6.8, 37.0) | 42.5 (35.4, 49.9) |  | 48.2 (33.3, 63.5) | 47.3 (42.0, 52.6) |
| Economical developed | 32.6 (15.9, 55.3) | 42.2 (35.1, 49.6) |  | 45.2 (30.5, 60.7) | 48.4 (43.1, 53.8) |
| Parental diabetes | 5.5 (1.3, 20.3) | 16.3 (10.2, 25.0) |  | 29.0 (16.1, 46.7) | 22.1 (17.9, 27.0) |
| Junior high education or more | 36.9 (18.8, 59.6) | 57.8 (50.1, 65.2) |  | 58.7 (43.1, 72.8) | 58.5 (53.0, 63.7) |
| Current smoking | 14.8 (3.4, 46.1) | 3.5 (21.3, 34.8) |  | 16.3 (7.0, 33.3) | 20.6 (16.3, 25.7) |
| Current drinking | 13.1 (4.0, 35.1) | 28.5 (22.2, 35.6) |  | 16.3 (7.3, 32.4) | 24.9 (20.3, 30.1) |
| Physical activity, MET, h/wk | 78.3 (41.8, 114.9) | 59.0 (48.0, 70.0) |  | 59.8 (43.7, 75.9) | 51.5 (44.9, 58.1) |
| BMI, kg/m^2^ | 25.2 (23.8, 26.5) | 27.2 (26.4, 27.9) |  | 25.2 (24.2, 26.3) | 26.6 (26.3, 27.0) |
| Waist circumference, cm | 85.3 (81.5, 89.1) | 90.2 (88.4, 92.1) |  | 85.3 (82.4, 88.1) | 90.0 (89.0, 91.1) |
| Fasting plasma glucose, mmol/L | 6.8 (6.3, 7.3) | 7.3 (7.0, 7.6) |  | 7.5 (6.8, 8.3) | 8.3 (8.0, 8.6) |
| OGTT-2h plasma glucose, mmol/L | 11.8 (8.2, 15.5) | 11.5 (10.9, 12.1) |  | 7.4 (4.9, 9.9) | 9.2 (7.4, 11.0) |
| Hemoglobin A1c, % | 6.7 (6.2, 7.2) | 6.9 (6.7, 7.1) |  | 7.6 (7.0, 8.1) | 7.5 (7.3, 7.7) |
| Systolic blood pressure, mmHg | 148.3 (137.4, 159.1) | 149.4 (145.0, 153.7) |  | 149.3 (143.2, 155.3) | 151.5 (149.1, 153.8) |
| Diastolic blood pressure, mmHg | 85.4 (81.3, 89.4) | 88.6 (86.7, 90.6) |  | 85.6 (82.1, 89.0) | 89.1 (87.8, 90.4) |
| Total cholesterol, mg/dL | 180.1 (169.1, 191.0) | 192.1 (185.3, 198.8) |  | 176.4 (170.3, 182.5) | 176.5 (171.3, 181.7) |
| Triglycerides, mg/dL | 101.0 (92.6, 109.4) | 234.7 (200.1, 269.3) |  | 97.5 (89.1, 105.9) | 235.9 (210.8, 260.9) |
| LDL-C, mg/dL | 104.3 (97.9, 110.6) | 112.2 (107.2, 117.1) |  | 98.7 (93.3 104.1) | 98.8 (95.0, 102.7) |
| HDL-C, mg/dL | 54.1 (51.1, 57.2) | 38.9 (37.2 40.6) |  | 52.2 (49.9, 54.5) | 36.4 (35.5, 37.4) |

Data are weighted means (95% CIs) for continuous variables, and weighted percentages (95% CIs) for categorical variables.

Abbreviations: MET, metabolic equivalent; BMI, body mass index; OGTT, oral glucose tolerance test; LDL, C, low, density lipoprotein cholesterol; HDL, C, high, density lipoprotein cholesterol.

**Figure S1. Prevalence, awareness, treatment and control of hypertension and dyslipidemia by obesity status in each glycemic group**


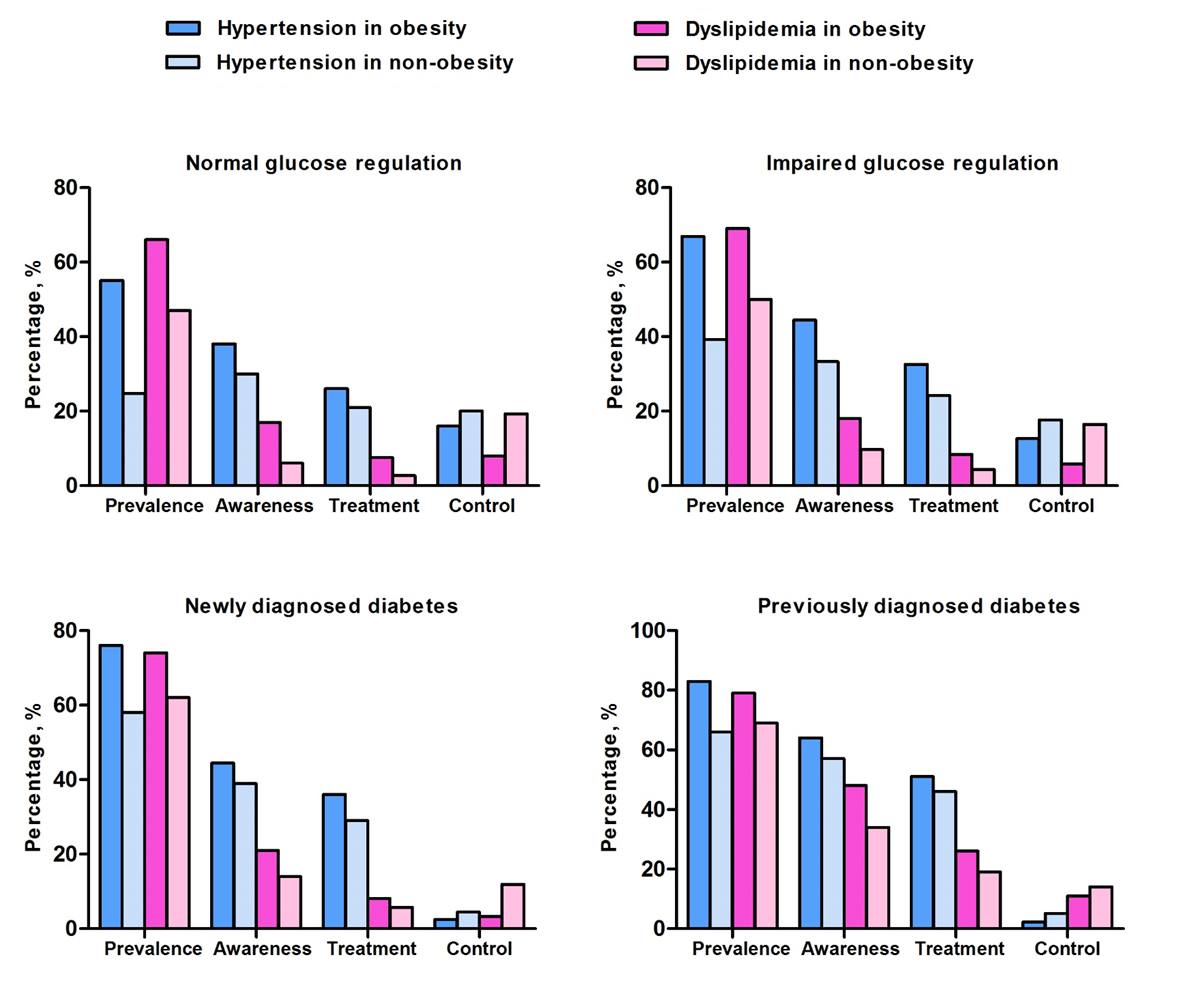

Supplement: Additional file 1: Table S1. — Characteristics of Chinese adults with controlled vs. uncontrolled hypertension in newly-diagnosed and previously-diagnosed diabetes. Table S2. Characteristics of Chinese adults with controlled vs. uncontrolled dyslipidemia in newly-diagnosed and previously-diagnosed diabetes. Figure S1. Prevalence, awareness, treatment and control of hypertension and dyslipidemia by obesity status in each glycemic group. [file 12933_2015_191_MOESM1_ESM.doc]
